# Supplementary material for: Are carbon emissions trading and green financial instruments synergistic? -Comprehensive quantitative research based on content analysis
Source: PLoS One. 2024 Mar 7;19(3):e0298601. doi: 10.1371/journal.pone.0298601 (PMC10919601; doi:10.1371/journal.pone.0298601)
Supplement: S1 Data — (ZIP) [file pone.0298601.s001.zip › Data/地级市Z_F工作报告.zip!/░▓╗╒/║╧╖╩╩╨/└┤╫╘╣1⁄2╓┌║┼í╛┬φ┐╦╩2╛▌═°í┐_2006.docx]

# **2006年政府工作报告**

发布时间：2006-01-31 08:08

【字体：[大](http://www.hefei.gov.cn/public/1741/javascript:void(0);) [中](http://www.hefei.gov.cn/public/1741/javascript:void(0);) [小](http://www.hefei.gov.cn/public/1741/javascript:void(0);)】

各位代表：

　　现在，我代表市人民政府，向大会作政府工作报告，请连同《合肥市国民经济和社会发展第十一个五年规划纲要(草案)》一并审议，并请市政协委员和其他列席人员提出意见。

****一、“十五”期间回顾和2005年的政府工作****

　　“十五”是我市改革开放以来经济社会发展最好的时期。5年来，在省委、省政府和市委的正确领导下，全市人民抢抓机遇，开拓奋进，提前完成了预定的主要目标任务，改革开放、经济建设和社会发展取得了巨大成就。

　　——综合经济实力跃上新台阶。5年来，经济发展呈现逐年加快之势，地区生产总值年均增长16.5%，占全省比重由2000年的13.2%提高到15.9%，综合实力在全国百强城市中由39位提升到31位。人均生产总值达到2315美元，比2000年翻一番多。财政收入年均增长25.4%。累计完成全社会固定资产投资1424.6亿元，是“九五”的2.9倍。经济结构调整成效明显，三次产业比例由10∶44∶46调整为6.2∶44.8∶49;工业经济快速发展，增加值年均增长14.6%;建筑业发展势头强劲，总产值年均增长35.4%;县域经济超常发展，肥西、肥东和长丰县在全省位次分别前移14位、3位和8位，农业规模化、优质化、产业化趋势明显加强;现代服务业方兴未艾，旅游业迅速崛起，全省旅游中心地位初步确立。物流、会展经济和中介服务蓬勃兴起，金融、保险、邮政、电信等保持了良好发展势头。

　　——体制创新和对外开放取得重大进展。国有资产管理体制改革不断深入，国有资本运营体系基本建立。国有企业改革取得丰硕成果，一批重点骨干企业成功实施资产重组;国有大中型企业初步建立现代企业制度，新增上市公司3户;国有、集体中小企业“双退”改革以及企业剥离办社会职能等配套改革基本完成。投融资体制、财税体制改革稳步推进，公共财政框架体系逐步健全。扎实推进农村税费改革，全面取消了农业税，农民负担大大减轻。成功实施区划调整和城市管理体制改革，城区发展空间得到拓展，社区功能不断强化。科技、教育、文化、卫生体制改革继续深化。行政管理体制改革和事业单位改制稳步推进。对外开放成绩显著，累计利用外资13.11亿美元，比“九五”增加40%;引进境内资金459亿元。3大开发区建设迈出新步伐，综合实力显著增强。新建并壮大一批县区工业园区，成为县(区)域经济快速发展的重要载体。外经外贸持续快速增长，地区进出口总额年均增长17%。科技兴贸取得重要进展，在全国20个科技兴贸重点城市中的位次前移4位。

　　——城乡面貌发生显著变化。坚持科学规划，加快城乡建设步伐。组织编制了城市近期建设规划和城市战略发展规划，城市分区功能定位进一步明确。立足“改造提升老城区、加快建设新城区”，老城区通过实施“打通断头路、整治小街巷、改造旧小区、开展大拆违”，整体面貌大为改观;高标准、高起点建设政务文化新区，基础框架及部分主体工程基本建成。建成宁西铁路(合肥至西安段)和合安、合徐高速公路，实现对外交通快速对接。农村路网逐步完善，实现乡乡通柏油路、村村通砂石路。

　　完成大房郢水库建设以及南淝河肥东段、巢湖大堤、瓦埠湖保庄圩综合治理等一批重点项目，中心城区百年一遇、农村20年一遇的防洪保安体系基本形成。城乡电网逐步完善，供水、供气、供热等保障能力不断提高。创建“国家卫生城市”、“国家环保模范城”活动深入推进，龙泉山垃圾处理场、望塘和朱砖井污水处理厂等一批环保设施相继建成，环境质量继续改善。新建包河大道、南淝河沿线绿化以及徽园、野生动物园等一批园林景观。完成退耕还林等绿化任务，新增成片造林面积51.4万亩，全市森林覆盖率提高到14.8%，城市建成区绿化覆盖率达37%。环城水系综合治理工程获得“中国人居环境范例奖”。土地管理不断强化，在全省率先建立土地储备交易制度、经营性用地“招拍挂”制度，土地利用效率明显提高。文明创建工作深入推进，实现稳定的良性循环。以实施“四把工程”为重点，加快江淮分水岭地区综合治理，农村薄弱地区的生产生活条件大为改善。村镇规划建设步伐加快，实施了草危房改造和村庄综合整治，村容村貌有了较大改观。

　　——社会事业实现跨越式发展。坚持经济社会协调发展，持续加大各项社会事业的政策支持和资金投入。科技事业取得长足进步。连续5年举办中国·合肥高新技术项目-资本对接会，连续4次获得“全国科技进步先进市”称号，成为全国首个科技创新型试点市。成功加入世界科技城市联盟。科普教育成效显著，全民科学文化素养明显提高。信息技术应用领域逐步扩大。教育在改革中发展。义务教育阶段均衡发展进程加快，高中段普及率迅速提高，优质教育资源不断增加。职业教育、民办教育规模显著扩大。整合高教资源，成功组建合肥学院。确立了农村义务教育“地方负责、分级管理、以县为主”的管理体制。农村中小学危房改造成绩显著，办学条件不断改善。“文化强市”战略初显成效，文艺创作和演出成果丰硕，文化市场管理加强，文化产业蓬勃发展。市科技馆、图书馆等一批重点文化设施项目相继建成。农村广播电视覆盖率达到100%。竞技体育水平始终保持全省领先地位，群众体育社会化格局基本形成。城乡医疗救治体系、疾病预防控制体系和卫生监督体系逐步健全，肥西县新型农村合作医疗试点取得成功。人口增长得到有效控制，人口质量不断提高。国防教育、民兵预备役工作不断加强，实现争创全国双拥模范城“五连冠”。民族宗教工作扎实有效，人防、外事、侨务、对台、地震、气象、统计、档案、保密、妇女儿童、未成年人保护、老龄、残疾人等事业迈上新台阶。

　　——人民生活水平显著提高。城镇居民人均可支配收入和农民人均纯收入年均分别增长9.8%和10.2%;城乡居民人均储蓄存款余额年均增长22%。社会消费品零售总额年均增长17%，居民消费价格指数年均增长0.7%。消费结构加快升级，住房、汽车等高档消费需求迅速扩大，文化、休闲、旅游等消费成为时尚。城镇居民人均居住面积和农村人均生活用房面积分别比2000年增加3.8和4.6平方米。城乡劳动力就业领域得到拓展，农村劳动力外出务工大幅增加。下岗失业人员再就业工作成绩显著，受到国务院表彰。社会保险覆盖面不断扩大，城乡困难群体救助体系逐步建立。在全省率先建立“土地换保障”制度，维护被征地农民的合法权益。扶贫开发成效明显，农村贫困人口由7.4万人减少到3.7万人，低收入人口由16.5万人减少到4.55万人。广泛开展精神文明创建活动，市民综合素质和城市文明程度不断提高，蝉联“全国创建文明城市工作先进市”称号。

　　——民主法制建设得到加强。加快政府职能转变，政府宏观调控能力、统筹发展能力、公共服务能力和危机管理能力大大增强。坚持依法行政，自觉接受人大、政协和各民主党派、无党派人士及社会各界的监督。建立健全定期联系人大代表制度和政协议政会制度，充分尊重人大代表、政协委员的意见和建议，5年累计办结人大代表议案32件、建议897件，办理政协委员建议案6件、提案1816件。完善与工会的联席会议制度，重视发挥共青团、妇联等群众团体的积极作用。加强基层民主政治建设，深入开展政务公开、企务公开、村务公开，成功进行了村民委员会换届选举和社居委调整工作。建立和完善了市长热线电话制度、人民建议征集制度、市长接待日制度、新闻发言人制度、社会听证制度、法律顾问制度，开通市长电子信箱，决策科学化和民主化不断增强。深化行政审批制度改革。组建行政服务中心，实行一个窗口对外、一条龙服务。分4批取消行政审批项目222项，合并下放审批191项。积极贯彻《行政许可法》，取消行政许可事项33项。完成“四五”普法教育各项任务。开展“平安合肥”创建活动，构建全方位的社会治安防控体系和安全生产监管体系。深入开展共产党员先进性教育活动，大力推进政府机关效能建设。建立健全政风评议和行风评议制度，综合运用审计、监察等监督手段，促进政风建设和廉政建设，投资发展环境明显改善。

　　2005年是“十五”最后一年，我们坚持以改革开放为动力，以加快发展为主题，全面落实科学发展观，实现经济社会快速健康发展，全市呈现出经济快速增长、社会和谐稳定、人民安居乐业的良好局面。

　　(一)国民经济快速增长。全年实现地区生产总值853.6亿元，增长16.9%;实现财政收入130.88亿元，增长24.2%，其中地方财政收入57.64亿元，增长28.3%;城镇居民可支配收入9684元，增长12.5%，农民人均纯收入3207元，增长11%。全社会固定资产投资495.3亿元，增长36.4%;社会消费品零售总额324.4亿元，增长15.1%。工业经济稳定增长，效益同步提高，规模以上工业企业实现总产值843亿元，工业增加值263.7亿元，增长23.5%;工业经济效益综合指数达207，提高近40个百分点。8大重点产业支撑作用进一步增强，实现产值占全市工业总量的78.2%。城郊型农业快速发展。种植业结构进一步优化，瓜菜果种植面积迅速扩展，无公害蔬菜基地面积达30万亩，无公害农产品发展到50个。在全国率先建立起职责明确、运转灵活、工作有效的动物防疫体系和畜禽产品质量安全监管体系，有效地防范了禽流感，养殖业规模继续扩大、效益持续增长，受到省委、省政府和国务院督查组充分肯定。农业产业化快速推进，龙头企业实力进一步提升，新增省级龙头企业7个，超亿元企业达到10家。成功举办了第3届中国·合肥苗木交易大会。第三产业实现增加值418亿元，增长16%。全年实现旅游总收入35.8亿元，增长22.5%。

　　(二)改革开放全面推进。国有大中型企业资产重组取得新进展。荣事达与美的集团、美菱股份与长虹电子实行强强联合，合铝集团、芳草日化和神马电工等一批企业实现重组。合钢、氯碱、四方集团等企业主辅分离、辅业改制工作积极推进。生产经营类事业单位改革以及党政机关与所办经济实体和直接管理企业脱钩改制工作步伐加快。政府非税收入管理改革、上市公司股权分置改革、农村信用社改革试点开始启动。大力推进国有产权进场交易，全年实现交易额突破20亿元。深化土地有偿使用制度改革，全年入库土地出让金近15亿元。城市管理体制改革取得新突破，行政执法改革全面推开;农村综合改革试点顺利推进，乡镇清财工作全面完成。强化目标责任制，突出重点领域和重点地区，广泛开展形式多样的招商活动，招商引资成效明显。全年新批外商投资企业91户，合同利用外资3.45亿美元，增长29.7%;实际利用外资4.07亿美元，增长28.7%。引进境内资金172亿元，增长40.7%。3大开发区和县区工业园区共引进项目544个，完成固定资产投资200亿元。天威保变工业园、生命科技园、宝业住宅产业化基地、伊利乳业工业园等一批重大项目开工建设。对外贸易快速增长，完成海关进出口总额41.8亿美元，增长19.2%，其中出口总额27.94亿美元，增长27.6%。市属企业完成进出口总额17.85亿美元，增长35.5%，其中出口11.79亿美元，增长60.3%。完成国际经济技术合作营业额2.41亿美元，增长114%。

　　(三)城乡建设稳步推进。开展了新一轮城市总体规划、土地利用总体规划修编工作。合六、合淮阜高速公路建设及二环路改造按计划推进。芜湖路、飞龙路、铜陵路桥等工程竣工。城市供水、供电、供气、供热和公交等公用设施建设顺利推进。蔡田铺污水处理厂和二里河上游综合改造工程正式启动，环城水系中水回用基本实现全线贯通。城市环境综合整治继续深入，“创模”工作通过省级考核验收。环城公园和芜湖路灯饰亮化工程竣工。推进防洪保安工程和农田水利基本建设，完成了瓦埠湖蓄洪区一、二期工程和丰乐河18公里河段整治。新开工农村公路国债及通达项目16个，“村村通混凝土路工程”开始实施。城区改造小街巷106条，新增园林绿地213块，新建改建公厕37座，整治脏破建筑233幢。园林旅游项目建设步伐加快，完成“三园”改造升级工程，三国遗址公园建设进展较快，包公园、徽园被评为国家4A级旅游景区。开工建设经济适用住房50万平方米，改造危旧房60万平方米。小城镇建设步伐加快，肥东县撮镇镇跻身全国首批发展改革试点镇。

　　(四)科技和社会事业蓬勃发展。全面启动国家科技创新型试点市建设，出台并实施《试点市工作方案》，示范区已有7个项目开工建设。成功举办第5届高新技术项目-资本对接会暨中国专利20年成果展。高新技术产业快速发展，全年实现总产值、技工贸总收入分别增长31%、30%。大力推进科技兴农，组织实施粮食丰产科技工程和“农业科技专家大院”项目。“1124”信息化工程加快推进，建成全市统一电子政务专网，联合审批系统、视频会议系统等70多项电子政务项目顺利推进，农业信息网不断完善。各类教育协调发展。义务教育成果得到巩固，全市小学、初中入学率、升学率保持较高水平。进城务工农民子女义务教育阶段入学问题初步得到解决。普通高中和职业教育协调发展，普职比趋于合理。文化事业不断发展。成功举办第16届国际美术大会。专业文艺创作精彩纷呈，群众性文化活动蓬勃开展。长江剧院重建、城隍庙修复、李府东扩工程竣工，赖少其艺术馆建成开馆。繁荣和发展哲学社会科学，理论研究有所突破和创新。竞技体育和全民健身工作取得新成绩。动工兴建市体育中心、广电中心和文化艺术中心。市疾病控制中心等一批卫生建设项目基本建成。社区卫生服务网络建设加快推进，肥东和长丰县列入国家和省农村新型合作医疗试点县。开展出生人口性别比综合治理，全面实施农村部分计划生育家庭奖励扶助制度。认真落实优抚安置政策，“双拥“工作整体水平继续提高。国防教育、后备力量建设扎实有效。人防工程建设面积大幅度提高，市级人防指挥所工程投入使用，荣获全国“人民防空先进城市”称号。《合肥市志》续修工作进展顺利。防震减灾工作得到加强。

　　(五)文明创建扎实开展。加强社会主义精神文明建设，提炼和倡导“城市精神”，公民道德教育、诚信建设、未成年人思想道德教育得到加强。实行任务分解和“双月推进计划”，深入开展全国文明城市创建活动，在全省考评中名列第一。开展声势浩大的查处违法建设工作，共拆除各类违法建设1000多万平方米，赢得了全市人民的积极拥护和社会各界的普遍赞誉。通过“大拆违”，拓展了发展空间，改善了城市形象，锻炼了干部队伍，提升了政府的公信度。结合“大拆违”，深入开展城市出入口、农贸市场等专项整治，重点区域的脏乱差现象明显改观。市、区、街道3级环卫督查网络初步建立，环境卫生管理工作进一步向城郊结合部、居民小区内部延伸。加强环卫基础设施建设，新建、改建生活垃圾转运站22座。

　　(六)城乡社会和谐稳定。积极推进就业再就业和社会保障工作，全年城镇新增就业岗位5.8万个，城镇登记失业率4.4%。大力拓展“组织起来就业”新模式，通过开发社区就业岗位、建立“百帮“创业基地、托底安置“4050”人员等多种措施，解决了一大批下岗失业人员的就业问题。调整完善社保政策，激发了各类职工参保的积极性，全市社会保险5项险种参保人数较上年末增加25万人，其中私营企业和个体工商户新参保人数超过历年总和。就业和社会保障工作服务平台进一步完善，全市所有街道、乡镇全部建立了企业退休人员管理服务所(站)，“退管”服务率达80%。完善最低生活保障制度，初步实行“分类施保”。在全市范围内开展了困难群体大排查，实施教育、医疗、住房等多项救助措施，保障了低收入群体的基本生活。组织实施扶贫开发“整村推进”工程，加大重点贫困村的脱贫力度。加强社会治安综合治理，严厉打击各类刑事犯罪，加大消防、交通安全等专项整治力度，连续3届被评为“全国社会治安综合治理工作优秀城市”，并获得首届“长安杯”。以贯彻新修订的《信访条例》为契机，依法整顿和规范信访秩序，在全省率先开展了党政领导干部开门接访、带案下访活动，有力化解了社会矛盾，密切了干群关系，全市信访稳定形势明显好转。强化安全生产管理，加大专项整治力度，安全生产形势保持稳定。全面加强城乡市场监管，坚决打击制假售假、非法行医等违法行为，强化文化市场、食品药品安全管理，维护良好的市场秩序。

　　回顾过去5年的工作，成绩来之不易。这是省委、省政府和市委正确领导的结果，是全市人民齐心协力、迎难而上、奋力拼搏的结果。在此，我谨代表市人民政府，向全市广大工人、农民、知识分子、干部、驻肥解放军指战员、武警官兵和政法干警，向各民主党派、工商联、人民团体和各界人士，致以崇高的敬意!向所有关心支持合肥改革开放与现代化建设的海内外朋友，表示衷心的感谢!

　　在总结“十五”取得的成绩同时，我们也清醒地认识到，当前经济社会发展中还存在一些突出问题和深层次矛盾：城市建设管理尚有很多亟待解决的问题;工业总量仍不够大，辐射带动能力不强;对外开放领域不宽、层次不高，招商引资总量不大;城乡二元结构矛盾仍然突出，农村经济发展相对滞后;就业和社会保障压力大，部分群众生活存在困难;政府效能建设、投资发展环境等方面还存在不少差距。“十一五”期间，必须采取有效措施，努力加以解决。

****二、“十一五”期间经济社会发展的总体目标和主要思路****

　　“十一五”时期既是十分难得的“黄金发展期”，又是不可忽视的“矛盾凸显期”。从发展机遇看，世界经济将继续保持较快发展，国际产业资本转移的大趋势不会改变;我国经济仍处在新一轮上升周期，国家积极实施中部崛起战略，将在发展政策、重大项目布局等方面向中部地区倾斜;省委、省政府明确提出中心城市带动战略和打造省会经济圈，对合肥的支持力度越来越大;作为首个国家科技创新型试点城市，将更加有利于借助创新的动力发挥后发优势;经过多年的积累，我市已经基本具备了跨越式发展的基础和条件，全市上下奋力崛起、勇当先锋的氛围日渐浓厚，将为今后的发展提供强大动力。从面临挑战看，国际竞争、国内区域竞争和产业竞争压力加大;我市经济社会发展中存在的深层次矛盾和问题将相互交织，形成更加复杂的制约因素。因此，我们必须抢抓一切发展机遇，主动应对各种挑战，积极破解各种难题，奋力实现超常规发展。

　　“十一五”我市经济社会发展的指导思想是：以邓小平理论和“三个代表”重要思想为指导，深入贯彻落实党的十六大和十六届五中全会精神，按照省委、省政府的战略部署，以科学发展观统领城乡经济社会发展全局，以提升经济总量、增强经济实力、提高人民生活水平为主线，大力实施工业立市、县域突破、创新推动、东向发展和可持续发展战略，推进全市经济社会实现跨越式发展，在安徽崛起中勇当先锋，在中部崛起中争先进位，为在全省率先实现全面建设小康社会、率先基本实现现代化奠定坚实基础。

　　“十一五”我市经济社会发展的总体目标建议为：地区生产总值年均增长15.5%，力争18%;财政收入年均增长15%;城镇居民可支配收入和农民人均纯收入年均分别增长11%和11.5%;全市万元生产总值综合能耗比2005年降低20%。经过5年奋斗，把我市基本建设成经济更加发达、科教更加进步、文化更加繁荣、社会更加和谐、生态更加良好、人民生活更加殷实的小康社会。

****实现上述目标，在实施“十一五”规划中，必须把握好以下几个方面：****

　　——优先发展先进制造业，强化现代服务业支撑力，全面提升重点产业竞争力。强化工业主导地位，集中优势资源，实施高强度投入，实现跨越式发展，基本形成技术高新化、产业集群化、信息化、标准化、资源集约化的现代制造业体系，建成国内重要的先进制造业基地和高新技术产业基地。立足现有产业基础和比较优势，增强支柱产业竞争优势，突出重点产业支撑作用，做大高新技术产业规模。调整优化产业布局，推动产业集聚。围绕龙头企业，培育中小企业集群。加快县域工业化进程，以工业园区为主要载体，围绕城市支柱产业、重点企业，加强协作，错位发展，培育和形成各具特色的配套产业。积极培育铺天盖地的民营企业，大力推进全民创业活动。强化现代服务业对工业的支撑作用，以发展区域性要素市场中心为突破口，大力发展金融、物流、会展以及文化、旅游、中介服务等现代服务业，促进服务业和制造业的互动发展。

　　——统筹城乡发展，加快县域经济突破，建设社会主义新农村。以发展为目标，以提高农民收入为核心，坚持以城带乡、以工促农，促进城市生产要素、基础设施向农村延伸，实现县域经济新突破。在强化县域工业的同时，推进农业产业化，发展特色经济、高效农业和无公害农产品，促进农业结构优化升级。培育综合性农产品批发市场及各类专业合作经济组织、行业协会，提高农村经济组织化、集约化、市场化程度。围绕改善农村生产生活条件，强化农村基础设施建设。分类梯次推进城镇化，提升城镇综合承载能力，逐步形成以城市为核心、县城为副中心、中心镇为骨干、中心村为基础的城镇体系。按照“生产发展、生活宽裕、乡风文明、村容整洁、管理民主”的要求，建设与现代化大城市相适应的社会主义新农村。

　　——以科技创新为先导，探索创新型城市发展道路，转变经济增长模式。以科技创新型试点市建设为契机，发挥科教资源优势，推进知识创新、技术创新、产业创新和制度创新，逐步把合肥建设成为全国著名的科技创新型城市。深入推进经济社会信息化，完善信息化网络基础设施建设，基本建成电子商务应用体系。以提高资源利用效率为核心，以节能、节水、节材、节地和资源、能源综合利用为重点，发展循环经济，建立健全节约型社会的体制和机制，形成节约型的增长方式。提倡清洁生产，倡导生态消费，保护自然环境，实现人口、资源、环境协调发展，可持续发展能力显著增强。

　　——实施“东向发展”战略，创新体制和机制，增强发展动力。立足“东向发展”，在思想观念、产业发展、要素流动、基础设施、体制机制等方面，实现与长三角等发达地区的对接和互动发展。

　　构建社会化、市场化、现代化的招商机制，扩大招商引资;完善贸易促进体系和指导服务体系，优化贸易结构，支持和引导地方企业开拓国内外市场，形成全方位、宽领域、多层次的大开放格局。坚持以改革促发展，破除束缚发展的体制性障碍。推进行政管理体制改革，加快转变政府职能，提高行政效能。深化国有企业和国有资产管理体制改革，引导非公有制企业制度创新和产业升级。发展资本市场和产权市场，培育土地、技术和劳动力等要素市场，完善现代市场体系。

　　——加强城市规划建设与管理，提升形象，完善功能，增强城市辐射力。按照“世界眼光、国内一流、合肥特色”的总体思路，以更高的起点、更大的手笔推进城市规划和建设。优化城市功能分区，加快构筑“1个主城、4个副中心、1个滨湖新区”的现代化大城市框架，逐步实现城市形态由单中心、高集聚向多中心、组团式转变，由“环城时代”向滨湖、临江城市的演变，努力打造独具魅力的现代化滨湖城市。加强城市基础设施建设，完成合宁、合武高速铁路、合肥铁路枢纽改造和合淮阜、合六、环城高速公路建设，建成合肥新港集装箱码头，基本建成合肥新机场。构建城市快速通道，建设大运量的快速公共交通系统，形成立体通畅的交通网络体系。完善水、电、热、气等设施，提高城市保障能力。围绕创建国家生态型园林城市、国家环保模范城市和人居环境最佳城市，实施高品位绿化，控制污染物排放，强化生态保护，改善环境质量，构建环境友好型社会。

　　——坚持以人为本，加强社会建设，努力构建和谐社会。统筹发展各项社会事业，整合社会各类资源，全面加强社会建设。推进教育事业发展，巩固扩大义务教育成果，大力发展职业教育，逐步建立各级各类教育协调发展的国民教育体系。实施“文化强市”战略，建设区域性文化中心城市。健全疾病预防控制体系、医疗救治体系和卫生监督管理体系，建立比较完善的城市社区卫生服务网络和新型农村合作医疗制度。完善就业服务体系，建立促进扩大就业的有效机制。更加注重社会公平，完善收入分配制度。健全与经济发展水平相适应的社会保障体系和城乡特殊困难群体社会救助体系，逐步提高最低生活保障和最低工资标准，认真解决低收入群众的住房、医疗和子女就学等困难问题。完善社会管理体系，构建党委领导、政府负责、社会协同、公众参与的社会管理新格局。建立健全安全生产长效机制，强化政府预警与应急管理体系建设，保障人民群众生命财产安全。加强社会治安综合治理，深入推进“平安合肥”建设。正确处理新形势下的人民内部矛盾，完善社会利益协调和社会矛盾排查调处机制。推进政府、企业、个人信用建设，建设“信用合肥”。加强民族宗教工作。加强国防后备力量建设，使国防建设与经济建设协调发展。围绕创建全国文明城市，深入开展群众性精神文明活动，形成积极健康、奋发向上的社会风尚，为推进跨越式发展营造良好的社会环境。

　　三、2006年的主要工作2006年是“十一五”开局之年，切实做好今年各项工作，对于顺利实施“十一五”规划具有十分重要的意义。我们要全面落实科学发展观，紧扣“大发展、大建设、大环境”这个主题，采取强力措施，突出工作重点，努力在推动跨越式发展上实现新突破。2006年经济社会发展的主要预测指标建议为：地区生产总值增长16%;财政收入增长15%;固定资产投资增长25%;实际利用外资增长23%;城镇居民人均可支配收入和农民人均纯收入分别增长11%和10%;城镇登记失业率控制在4.5%;人口出生率控制在12‰。围绕以上目标，着重抓好以下工作：

　　(一)全面实施“工业立市”战略，加快发展工业经济。认真落实《优先加快工业发展行动纲要》，出台《加快新型工业化发展若干政策》及目标考核奖惩办法，组织开展“加快工业发展年”活动。实施重点产业发展规划和专项规划，制定加快发展的政策和措施。支持支柱产业加速发展，突出发展汽车、装备制造、家用电器、化工、新材料、电子信息及软件、生物医药、食品及农副产品加工等8大重点产业，新增产值超百亿元工业企业1户、超50亿元2户。大力培育新的经济增长点，鼓励和引导各类资本投入工业，确保完成工业投资120亿元，力争完成140亿元，重点推进“121”工程，实施好化工企业搬迁改造、联合利华工业园、江汽轿车、美的、天威保变、乐凯工业园、佳通全钢子午胎等一批重大项目，提升工业整体竞争力。出台《工业产业空间布局导向目录》，统筹城乡产业布局，引导产业合理分工。加强中小企业、民营企业社会服务体系建设，扶持一大批“专、精、新、特”中小企业发展。坚持走新型工业化道路，以信息化带动工业化，依靠科技创新，不断提高企业核心竞争力。大力发展循环经济，广泛推广清洁生产模式，突出抓好钢铁、化工、建材等高能耗行业和重点企业的节能工作，加快节能省地型住宅产业、农作物秸秆综合利用等工程建设。

　　(二)大力实施县域经济突破，推进社会主义新农村建设。围绕中央建设社会主义新农村的总体要求，以规划示范、村镇建设、基础设施、信息网络、公用事业和文明创建为抓手，全面启动新农村建设。按照实施县域突破战略的要求，加快发展县域经济，不断壮大县域经济实力。坚持以工业化带动农业产业化，以先进制造业、城市配套工业、农副产品加工业为主攻方向，做精、做强城市配套服务业和特色经济，形成各具特色的主导产业，构建县域经济增长极。继续推进江淮分水岭地区综合治理。加快农业结构调整，优化农业布局，推进规模经营，加快发展优质、高效、设施农业。推进集约型养殖小区建设，组织实施“千场百区”工程，进一步提升养殖业在农业中的比重;坚持产业为先、生态并重，提升苗木花卉产业，打造全国重要的绿化苗木生产基地;推进设施栽培小区建设，扩大专业菜地、反季节和精细蔬菜基地面积，做大做强西甜瓜、草莓等产业。大力培育龙头企业，实施农产品“创牌工程”，提高农产品竞争力和市场占有率。完善农村社会化服务体系，充分发挥农村致富带头人和农村经纪人的作用，提高农业和农村经济组织化程度。大力发展劳务经济，加强组织引导和职业教育培训，加快农村劳动力向二、三产业转移，促进农民持续增收。加大扶贫开发工作力度，组织实施“整村脱贫”项目。推进城乡思想观念对接、体制机制对接、规划对接、设施对接、产业对接、市场对接和人才对接，加快城市道路、公交、供电、供水、供气、环保、信息、金融、商贸等服务设施向县域延伸。继续抓好农村水利建设，进一步强化防洪保安和节水灌溉能力。加快农村道路、饮水、电网、通讯等基础设施建设，认真实施“村村通混凝土路”工程。加快村镇规划建设步伐，重点推进发展改革试点镇和中心镇、中心村建设，积极推进农村社区建设。

　　(三)加快国家科技创新型试点市建设，增强自主创新能力。全面贯彻全国科技大会精神，以增强自主创新能力为核心，积极探索建设创新型城市的新路子。认真分解落实科技创新型试点市工作方案，建立健全工作协调机制和服务机制，落实政策支持措施，出台目标考核奖励办法。大力支持企业技术创新体系建设，培育和发展25家科技创新型企业，新增3家省级以上工程(技术研究)中心，建立14家市级企业技术中心和10家省级制造业信息化示范企业;积极探索产学研有效结合的新机制，鼓励和支持科研人员领头创办企业，鼓励和支持科研单位走产业化发展的路子，努力形成以科技合作项目为主要内容的多层次、全方位合作形式;建立多元化的科技创新投融资体制，设立试点市发展专项资金和科技型中小企业技术创新基金;加快高新技术产业发展，力争全市高新技术产业产值达到600亿元，技工贸总收入达到570亿元，增幅达到27%以上;加快信息产业与其他制造业的融合，发展具有自主版权的应用软件，推进信息技术在各领域的广泛应用;继续办好中国·合肥高新技术项目———资本对接会，扩大合肥高新技术产权交易市场;推进试点市示范区规划建设，加大项目引进和投资力度，争取新开工10至15个项目，完成投资10亿至15亿元，推动示范区尽快成规模、出形象。加大创新型试点市宣传和推介力度，加强科学普及和人才工作，形成浓厚的创新氛围。

　　(四)加强城市规划建设和管理，进一步完善城市功能。围绕建设现代化滨湖城市，按照“新区开发、老城提升、组团展开、整体推进”的思路，加快新一轮城市总体规划和土地利用总体规划修编，做好中心城区、副中心和各类专业规划、区域规划的编制，形成较为完善的城乡规划审批与管理体系，尽快扭转规划滞后及管理不到位的局面。积极争取将合肥纳入长三角发展规划，推进合肥与长三角地区的一体化发展，为打造省会经济圈创造良好条件。启动滨湖新区的规划与建设。以建设综合性的交通枢纽为重点，加快完善城市基础设施。积极推进合宁、合武高速铁路、合肥铁路枢纽改造和合淮阜、合六高速公路以及合肥新机场、合肥新港集装箱码头等一批重大工程建设，构建城市立体对外交通体系。城市规划区内要加快构筑快速交通网络，开工建设中心城区到3县的快速干道，尽快解决路网结构不合理、交通拥堵的问题。进一步加大老城区道路及成片改造的力度，重点向薄弱区域倾斜。提升改造城市主干道，打通一批“断头路”，改造城市出入口;继续做好道路节点交通渠化工程，加快社会停车场建设;切实做好“城中村”、危旧房、棚户区、小街巷改造工程的规划和实施。加快经济适用房建设，抓好廉租住房工作，增加中低价位、中小户型普通商品房供给。推进政务文化新区建设，抓好文化艺术中心、广电中心、少儿活动中心等项目建设，完成体育中心工程。加快公用事业改革和发展，继续推进水、电、气、热等市政设施建设。规划建设生态型园林城市，抓好高压走廊绿化、拆违建绿及百块绿地建设。初步建成三国遗址公园和花冲儿童主题公园。继续深入开展“创模”活动，力争通过国家级考核验收。开工建设望塘污水处理厂二期，竣工蔡田铺污水处理厂一期工程及配套管网。开展十五里河、四里河、二十埠河、板桥河等综合治理。规划建设新殡仪馆。进一步加强城市管理，开展市容、交通、农贸市场等综合整治，着力解决脏乱差、行路难等突出问题。继续坚定不移地推进“大拆违”，巩固成果，防止反弹，并以此为契机，建立健全城市精细管理、长效管理的新机制。加强土地管理和基本农田保护，开展农村宅基地整理，节约和集约利用土地，最大限度地发挥土地资源的基础作用。

　　(五)加快传统商业改造升级，大力发展现代服务业。推进商业结构战略性调整，培育主业突出、竞争力强的龙头企业，重点发展壮大百大集团等销售额超20亿元企业集团和专业批发市场。鼓励优势企业实施规模扩张，支持企业进行跨行业、跨地区、跨所有制资产重组，引导各种资本参与流通企业改组和新型商业服务业发展。积极支持以连锁经营、电子商务等为主要特征的现代流通方式，改造提升传统商业。加快中央商务区规划建设步伐，增强辐射带动能力。加快各类专业市场、社区商业设施建设，深入推进“万村千乡”工程。大力发展新兴服务业，鼓励引导发展健身、娱乐等休闲服务和信息、咨询等中介服务。继续推进全省旅游中心城市建设。改造提升现有旅游景区，积极申报国家4A级景区。开工建设渡江战役纪念馆，修复刘铭传故居。大力发展会展经济，加大全国性会展的申办和具有地方优势和特色的品牌会展培育力度，不断提升展会规模和水平。建立健全房地产市场信息系统，保持房地产业健康有序发展。加大生产生活资料市场监管，推进食品、药品放心工程。

　　(六)深入推进体制改革，进一步提高对外开放水平。完善国有资产营运体系，提高国有资产营运效率和监管水平。加快以合钢集团等为重点的国有大中型企业战略性重组，完成公用事业、建筑安装等企业改革改制任务。按照分类指导原则，加快推进事业单位改革。全面完成生产经营类事业单位转企改革的扫尾工作。健全土地市场“招拍挂”、政府集中采购、产权交易、工程招投标等各项制度。积极开展“全民创业行动”，破除一切体制和机制障碍，完善创业激励机制，充分发挥各种创业潜能。全面落实促进民营经济发展的各项政策，引导民营企业加快制度创新、管理创新和技术创新，积极支持一批企业在优势产业和领域做大做强。推进农村综合改革，基本完成乡镇机构、农村义务教育和县乡财政管理体制改革任务。

　　加快实施东向发展战略，坚持资金、技术和人才一齐引，内资和外资一齐用，不断提升对外开放的层次、领域和水平。完善招商工作机制，改进招商方式，强化目标考核，扩大引资规模。建立一批专业招商队伍，对长三角等重点区域实行驻点招商。着力吸引大企业、大公司地区总部、研发机构落户合肥。进一步推进3大开发区体制和机制创新，不断增强吸引力，壮大企业规模。加快县区工业园区发展步伐，突出特色，完善功能，努力实现园区产业的提档升级。坚持科技兴贸，切实转变外贸增长方式，优化出口商品结构。继续加大合肥出口加工区的申报力度。加强对外经济技术合作，鼓励有比较优势的企业对外投资、承包工程和跨国经营。充分发挥外事、侨务等部门的纽带作用，扩大对外交往与合作。进一步做好对台工作，促进对台经贸往来与合作。加大外宣工作力度，不断提升合肥的对外形象。

　　(七)切实做好投资和财税金融工作，千方百计扩大融资渠道。完善财政增收机制，巩固壮大支柱财源，增强市级财政调控能力。坚持依法治税，强化税收征管。优化财政支出结构，重点支持“工业立市”、科技创新、公共服务、社会发展等方面的投资。坚持集中投入和重点扶持，逐步建立稳定的农业投入增长机制。深化财政改革，积极推行财政支出绩效评价，加快健全预算编制、执行与监督“三分离”的预算管理新模式。积极推进农村信用社改革，成立农村合作银行。进一步加大对银行信贷工作的支持，完善中小企业贷款担保制度，不断拓宽银政、银企合作领域。坚持“发展为上、投资为本”的理念，创新投资思路，逐级逐次细化责任，采取强力有效的措施与方法，积极争取各种可能的投入。围绕“十一五”发展规划，进一步加强投资项目的谋划和实施，细化项目责任与措施，做到储备一批、开工一批、在建一批。充分利用国内外资本市场和货币市场，更多地争取国外政府贷款、国债项目等各类资本和资金投入合肥的建设和发展。积极支持有条件的企业在境内外上市或发行企业债券，扩大直接融资规模。出台奖励政策，充分调动各类投资主体上项目、争资金、抓落实的积极性。

　　(八)高度关注民生问题，完善就业和社会保障体系。调整完善促进就业的优惠政策，建立城乡一体的劳动力市场，广开就业门路，挖掘就业岗位，新增就业5万人。鼓励劳动者自主创业和自谋职业，建立鼓励企业增加就业岗位的有效机制。继续推行“组织起来就业”模式，加强和规范百帮就业服务中心、劳务型公司建设管理，拓展百帮创业园的功能，使之成为就业创业基地、技能培训和实训基地。继续做好技能培训、创业培训与就业岗位的对接工作，分类做好高校毕业生、复转军人、被征地农民和进城务工人员的就业指导和服务工作。继续强化扩面征缴，完善社会保险“五险合一”征缴工作机制，基金征缴率保持在90%以上。加强市级统筹工作，完善社会保险政策。建立补充医疗保险制度，改进医保费用结算方式，实行网上结算和网上实时监控。完善街道社区“退管”工作平台建设，企业退休人员社区直管服务率达到90%以上。完成统一的社会保障卡发行工作，向社会提供方便快捷的信息查询服务。加大源头治理力度，健全工资支付和最低工资保障制度，落实农民工工资保障金制度，改善农民进城就业环境。以完善“分类施保”制度为重点，构建信息畅通、反应灵敏、运行高效的综合救助平台，切实保障困难群体的基本生活。

　　(九)加强精神文明建设，统筹发展社会事业。以全面提高人的现代素质和文明程度为目标，切实加强精神文明建设，努力在全社会形成共同的理想信念和精神支柱。继续推进全国文明城市创建活动，巩固扩大文明创建成果。认真做好中小学布局规划和建设工作，加大投入，积极改善薄弱学校、特殊教育、民族教育学校和进城务工人员子女定点学校的办学条件，落实农村义务教育杂费免收政策，促进义务教育均衡发展。加快职业教育发展，启动职业教育实训基地和技师学院建设项目，建立县区职业教育培训中心。积极做好危改资金管理工作，改善农村中小学整体办学条件。加大文化体制改革力度，促进文化产业发展。抓好精品文艺创作和演出，深入开展各种群众文化活动。加强文化市场和出版物市场管理，建设1至2个文化市场示范点。加大公共卫生服务网络建设力度，强化公共卫生安全体系。基本建成覆盖全市城区的社区卫生服务网络。优化农村卫生资源配置，加强乡镇中心卫生院建设。扩大新型农村合作医疗覆盖面，支持肥西县创建全国农村卫生工作示范县。积极申办全国第4届体育大会，参赛省第11届运动会，备战全国第6届城运会。继续实施农村部分计划生育家庭奖励扶助制度，强化出生人口性别比综合治理，全面启动关爱女孩示范市工作。加快国防动员指挥中心建设，大力推进全民国防教育、民兵预备役、国防动员和人防工作，不断提高双拥工作整体水平。完成全国农业普查工作。继续做好《合肥市志》的续修编纂工作，加强防震减灾综合能力建设，抓好民族、宗教、档案、保密、气象、广播电视、妇女儿童、未成年人保护、老龄等工作，积极发展残疾人事业。

　　(十)加强民主法制建设，维护城乡社会稳定。政府及各部门要坚决执行人大的各项决议、决定，认真负责地向人大报告工作，主动接受人大的法律监督;支持政协和各民主党派、工商联开展工作，主动接受其民主监督;关注社情民意，自觉接受广大人民群众和舆论的监督。认真办理人大代表议案、建议和政协委员提案，不断增强依法办事的能力。发挥职工代表大会、村民委员会、社区居民委员会的作用，完善基层自治组织和企事业单位的民主制度，保证人民群众依法行使民主权利。支持工会、共青团、妇联等人民团体开展工作，培育非政府组织，增强社会自治能力。增强立法的科学性和公开性，进一步提高地方性法规草案和政府规章的质量。全面推进“五五”普法教育工作，深入开展行业和基层依法治理活动，提高全社会法制化管理水平。加快发展律师、公证、仲裁等法律服务，扩大法律援助覆盖面，健全法律服务体系。深入推进“平安合肥”建设，强化社会治安综合治理，依法严厉打击刑事犯罪活动和敌对势力的破坏活动，确保“两抢一盗”等刑事案件发案数明显下降，增强广大人民群众的安全感。认真做好信访和人民调解工作，完善矛盾纠纷排查调处机制，化解人民内部矛盾。严厉查禁黄赌毒等社会丑恶现象，净化社会风气。高度重视安全生产工作，严格落实安全生产责任制，切实加强安全监管体系、应急救援体系、重大危险源监控体系建设，强化城乡公共安全管理，严防重特大事故发生。

　　各位代表，合肥正处于推进大发展、大建设、大环境，努力实现跨越式发展的关键时期，人民政府肩负着重大责任。我们要全面贯彻省委、省政府“两风”、“两情”建设的总体要求，坚决服从市委的统一领导，主动接受人大、政协的监督，深入开展“机关效能建设年”活动，大力推进“效能革命”，努力建设高效、务实、诚信、法治的政府。一是要创新工作思路和方法。加强学习与调查研究，解放思想，开拓创新，改进工作方式，适应新形势，破解新难题，寻求新突破。二是要加强政府能力建设，在改进管理经济方式的同时，更加注重社会管理和公共服务，增强处置突发事件、应对公共危机和营造优良环境的能力，不断提高服务能力与水平。三是要创新管理体制与运行机制。深化行政审批制度改革，创新审批方式，推进行政许可的集中办理、联合办理。继续抓好现有行政收费项目的清理，坚决取消不合理的收费项目，严禁搞搭车收费、有偿服务和强制性服务。严格控制各类检查、评比和达标活动。倡导办事讲求“效率、效益、效果”、“新事新办、特事特办、急事急办”等效能理念，强力推行限时服务制、两次终结制和特事特办制，严处有令不行、办事拖拉、吃拿卡要和态度刁蛮等行为。细化考核责任制，严格推行问责制，促进行政机关服务质量和服务水平的根本改善，努力把合肥打造成中西部地区乃至国内审批环节最少、办事效率最高的地区。四是要始终坚持依法行政。完善政府决策规则和程序，进一步推进政务公开，促进政府决策的科学化、民主化。深入贯彻国务院《全面推进依法行政实施纲要》，加快建设法治政府。强化行政执法责任制，明确界定并合理分解执法职权，完善执法评议考核机制，不断提高执法水平。五是要大力加强廉政建设。认真贯彻预防和惩治腐败的各项措施，加快建立教育、制度、监督并重的惩防体系。坚持和完善领导干部重大事项报告、述职述廉、民主评议、谈话诫勉、经济责任审计等制度，切实加强对重点环节、重点部门权力监督。严肃查处贪污、受贿等违纪违法案件，维护政府的良好形象。六是要努力建设诚信务实的政府。牢固树立执政为民的理念，想民之所想，急民之所需，千方百计把人民群众的根本利益维护好、实现好。强化信用意识，取信于民，不断提高政府的公信力。大力弘扬求真务实的精神和昂扬向上的风气，坚决反对官僚主义，力戒形式主义，狠抓各项工作的落实，用实实在在的工作业绩，换来合肥实实在在的发展，给人民群众带来实实在在的利益。

　　各位代表，实现合肥跨越式发展的目标宏伟，任务光荣而艰巨。让我们紧密团结在以胡锦涛同志为总书记的党中央周围，在省委、省政府和市委的坚强领导下，高举邓小平理论和“三个代表”重要思想伟大旗帜，深入贯彻党的十六大和十六届五中全会精神，坚定信心，抢抓机遇，奋力崛起，勇当先锋，为整体推进现代化大城市建设、实现全面小康社会的目标而努力奋斗!
